# Socio-demographic Predictors for Urban Community Disaster Health Risk Perception and Household Based Preparedness in a Chinese Urban City

**DOI:** 10.1371/currents.dis.287fb7fee6f9f4521af441a236c2d519

**Published:** 2016-06-27

**Authors:** Emily YY Chan, Janice Yue, Poyi Lee, Susan Shuxin Wang

**Affiliations:** Collaborating Centre for Oxford University and CUHK for Disaster and Medical Humanitarian Response (CCOUC), JC School of Public Health and Primary Care, The Chinese University of Hong Kong, Hong Kong S.A.R., China; Nuffield Department of Medicine, University of Oxford, Oxford, United Kingdom; FXB Centre of Health and Human Rights, Harvard University, Cambridge, Massachusetts, USA; Collaborating Centre for Oxford University and CUHK for Disaster and Medical Humanitarian Response, The Chinese University of Hong Kong, Hong Kong S.A.R., China; Collaborating Centre for Oxford University and CUHK for Disaster and Medical Humanitarian Response, The Chinese University of Hong Kong, Hong Kong S.A.R., China; Collaborating Centre of Oxford University and CUHK for Disaster and Medical Humanitarian Response (CCOUC)The Chinese University of Hong Kong

## Abstract

Objectives: There is limited evidence on urban Asian communities' disaster risk perceptions and household level preparedness. Hong Kong is characterized by high population density, and is susceptible to large-scale natural disasters and health crises such as typhoons, fires and infectious disease outbreaks. This research paper investigates the rates and predictors of urban community disaster risk perception, awareness and preparedness, at individual and household levels.

Methods: A randomized cross-sectional, population-based telephone survey study was conducted among the Cantonese-speaking population aged over 15 years in Hong Kong. Descriptive statistics were reported. A stepwise multivariate logistic regression analysis was conducted to determine the independent associations between risk perceptions, socioeconomic factors, household characteristics, and personal background.

Findings: Final study sample comprised of 1002 respondents with a 63% response rate. The majority of respondents (82.3%) did not perceive Hong Kong as a disaster-susceptible city. Half (54.6%) reported beliefs that the local population had lower disaster awareness than other global cities. Infectious disease outbreak (72.4%), typhoon (12.6%), and fire (7.1%) were ranked as the most-likely-to-occur population-based disasters. Although over 77% believed that basic first aid training was necessary for improving individual disaster preparedness, only a quarter (26.1%) of respondents reported participation in training.

Conclusion: Despite Hong Kong’s high level of risk, general public perceptions of disaster in Hong Kong were low, and little preparedness has occurred at the individual or household levels. This report has potential to inform the development of related policies and risk communication strategies in Asian urban cities.

## WHAT WAS KNOWN ABOUT THE SUBJECT

Densely-populated urban cities in Asia are among the highest at risk of natural disaster and health-related emergency, yet little is known about risk literacy and household preparedness in these settings. Findings in other populations suggest that communities are often ill-equipped to cope with disaster and evacuation, regardless of their susceptibility to threat. Household and individual preparedness is critical to the overall effectiveness and cost-efficiency of national disaster response strategy.

## NEW KNOWLEDGE THAT THE MANUSCRIPT CONTRIBUTES

This is the first investigation of household risk literacy and disaster preparedness in Hong Kong. In a randomized, population-based cross-sectional study of risk perceptions and knowledge, we found low levels of risk literacy and poor preparedness practices among the general public. The most commonly perceived potential hazards were infectious disease outbreak, typhoon and fire. Few respondents had prepared the necessary actions or materials for disaster, including completion of first aid training and stockpiling of non-perishable food and water, but most reported supplies of basic household medical supplies. Individuals with chronic health issues were more likely to have a long-term supply of medication available. Further public health strategies are needed to inform the population of likely hazards (such as extreme temperature events) and how they can best prepare for emergencies.

## INTRODUCTION

Highly populated cities in Asia are at greatest risk of emergency resulting from natural disasters.^1^ Recent large-scale disasters such as Typhoon Haiyan in the Philippines and volcanic eruptions in Indonesia have focused global attention on the catastrophic impact of natural disasters and the importance of disaster preparedness. Disaster preparedness plays a critical role in mitigating the adverse health effects of natural disaster. Preparedness is defined by United Nations International Strategy for Disaster Reduction (UNISDR)^2^ as knowledge, capabilities and actions of governments, organizations, community groups, and individuals ‘‘to effectively anticipate, respond to, and recover from, the impacts of likely, imminent or current hazard events or conditions’’. Preparedness efforts range from individual level activities (such as first aid training), to household actions (stockpiling of equipment and supplies), community efforts (training and field exercises), and governmental strategies (setting up early warning systems and contingency plans, the development of evacuation routes, and public information dissemination).

Perceived risk, disaster preparedness knowledge, prior disaster experiences and certain socio-demographic characteristics such as gender, age, education and family income have potential to affect emergency preparedness and related behaviors.^3^^,^^4^ Beyond socio-demographic variables, Botzen and colleagues found that less knowledge about the causes of flood events was associated with lower flood risk perception.^4^ Another Swiss study pointed out that individuals with experiences of previous floods had higher response efficacy and stronger intentions to take adaptive actions than those not previously exposed.^5^

Disaster preparedness has potential to mitigate the adverse human impacts of emergencies and disasters. Yet studies conducted in western communities have indicated that although the population attributed high importance to disaster preparedness, perceived preparedness did not necessarily translate to actual household preparedness and emergency response.^6^ In Asia, there is a major evidence gap in understanding how urban Chinese communities perceive natural disaster risk and the types of individual and household level preparedness adopted.

In addition, as defined by Petts et.al.,^7^ risk literacy was defined as the individuals’ underpinning of knowledge and concepts of uncertainty in risk assessment, and how they make sense of the health risk issues in particular. Hong Kong, an Asian urban, high-density community, may therefore act as a starting point for understanding community disaster health risk literacy and preparedness in Asia. Such knowledge is necessary to inform policy makers and emergency responders of the baseline level of preparedness in order to create effective policies and contingency plans. This study examines the profile and predictors of community disaster risk perception, awareness and household-based preparedness using a randomized cross-sectional population-based household telephone survey. The paper will also discuss how our findings may give insight to future disaster risk literacy and preparedness investigations.

## METHODS

**Study design and study population**

A cross-sectional, population-based telephone survey was conducted between May and June 2012. The study population was the non-institutionalized population aged 15 years or above residing in Hong Kong, including residents holding valid work or study visas. Exclusion criteria included i) non-Cantonese-speaking respondents; ii) overseas visitors holding tourist visas to Hong Kong; iii) 2-way permit holders from mainland China; and iv) those who were unable to be interviewed due to medical reasons.

Approval of the study protocol was obtained from the Survey and Behavioural Research Ethics Committee of The Chinese University of Hong Kong. The participants were briefed on the background, nature and purpose of the study. They were asked to give oral consent to participate at the beginning of the study and ensured their responses would be kept confidential.

**Instrument**

A structured questionnaire was constructed and used for data collection. The questionnaire consisted of 66 questions that aimed to collect the following information from respondent:

1. *Socio-demographic and background information*, including age, gender, district of residence, occupation and employment status, educational attainment, type of housing, household income, living with young children or elderly, experience in disasters and relief work (total 16 questions).

2. *General perception and risk literacy on disasters*, including perceived susceptibility of disasters in Hong Kong, perceived disaster awareness and preparedness of Hong Kong compared to other metropolis, risk perception of disasters that was perceived as most threatening, individual disaster risk literacy (total 33 questions).

3. *Disaster preparedness and training on first aid*, including questions on household disaster preparedness, perceived need of first aid training, experience in first aid training and characteristics of the first aid training received (total 16 questions).

4. *General risk perception*, including questions on risk ranking of a list of daily events such as natural disasters, diseases, daily risks, technological risks and environmental risks (total 1 question).

Interviews were conducted in Cantonese, the most common language of Hong Kong (spoken by 89.5% of population),^8^ and each took approximately 15-20 minutes to complete. The survey questionnaire was pilot tested in April 2012 (N=52) and refined based on the results of the pilot study.^9^

**Data collection**

A list of randomly generated telephone numbers was used to provide the study sample. The telephone interviews were conducted by trained interviewers. The telephone calls were made between 6:30pm to 10:00pm on weekdays and during daytime on weekends, to prevent over representation of the unemployed population. The subject undergoing the interview was chosen based on the “last birthday method”,^10^^,^^11^ in which the household member whose birthday was closest to the interview date was invited to participate in the survey.

**Statistical Analysis**

Descriptive statistics on the socio-demographic variables, perceived susceptibility of disasters in Hong Kong, perceived level of disaster awareness and preparedness of Hong Kong compared to other metropolitan cities, as well as individual and household level disaster preparedness was reported and the age-sex composition was compared to that of the recent Hong Kong Census data. Individual disaster preparedness was defined by having received first aid training while a proxy variable for household preparedness was calculated by summating the five variables of owning a first aid kit, having basic aids supplies, storing emergency food and water, having extra supplies of basic medication, and possessing a fire extinguisher. Univariate analysis was conducted, to identify significant independent socio-demographic variables with the disaster-related risk perceptions and disaster preparedness. Forward stepwise multivariate logistic regression was then applied on variables with at least a marginal statistical significance of p<0.05 in the univariate analysis in order to identify socio-demographic predictors for perceived susceptibility to disaster as well as actual level of disaster preparedness. All statistical analyses were conducted using PASW Statistics 18 for Windows (SPSS Inc., Chicago, IL, USA). Statistical significance was set at α=0.05.

## RESULTS

A total of 11,034 telephone calls were dialed during the data collection period. The numbers were called for a maximum of 5 times before being classified as unanswered. Among the numbers called, 2,790 were not answered and 6,653 were invalid. There were 1,591 persons who were eligible to participate in the study. However, among them 83 were not at home and 484 refused to participate; 22 accepted to participate in the survey but did not complete the interview. Hence, the final number of respondents who completed the survey was 1,002, and the response rate was 63.0% (1002/1591) (see Figure 1).

**Study flow of participants in the telephone survey:**
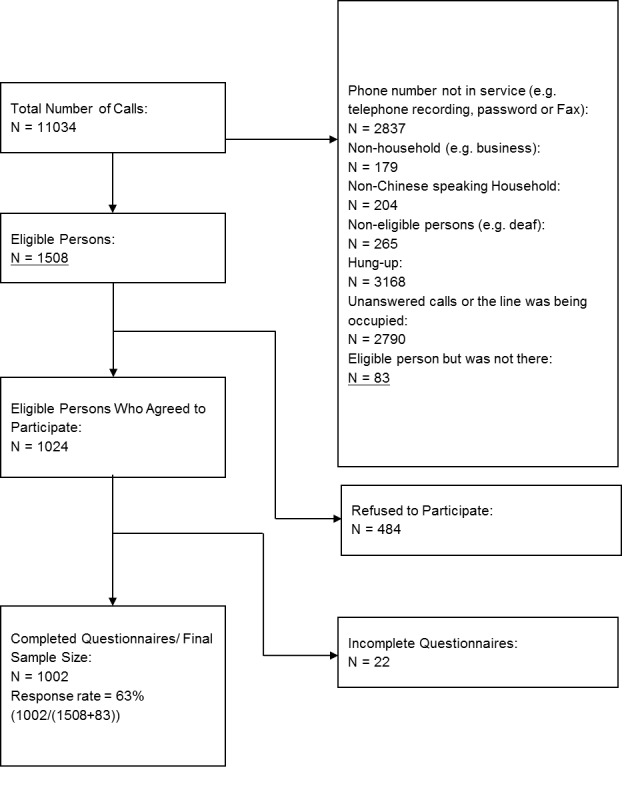

A total of 1,002 completed surveys were obtained from non-institutionalized, Cantonese-speaking Hong Kong residents aged 15 yrs and above during the survey period. Table 1 shows a comparison of the socio-demographic characteristics between the study population compared and the general population in Hong Kong in 2011.^8^ In general, the socio-demographic distribution of gender, population age structure and districts of the study population were comparable to the population data from 2011 Hong Kong Census.

**Figure:**
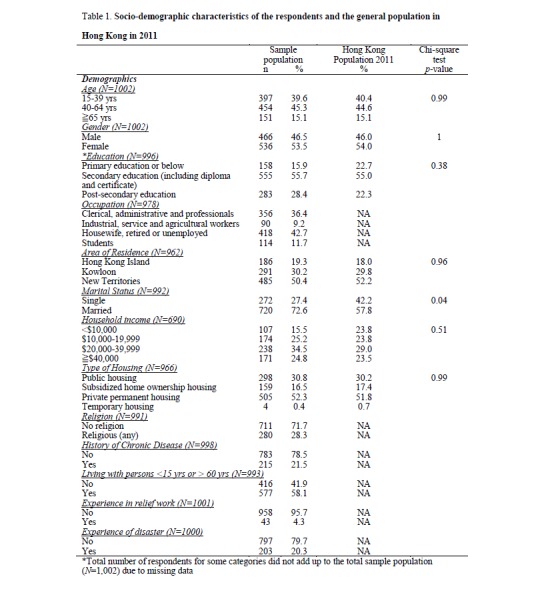
**Table 1. Socio-demographic characteristics of the respondents and the general population in Hong Kong in 2011**

**Perceived susceptibility to disasters** **, awareness and household preparedness** **of Hong Kong** **community**

The majority (87.2%, n=820) of respondents did not perceive Hong Kong to be susceptible to natural disasters. Infectious disease outbreak (74.0%, n=724), typhoon (12.9%, n=126) and fires (7.3%, n=71) were reported to be the most likely disaster threats to the population in Hong Kong. Of note, only 1.2% (n=12) of the study respondents perceived extreme temperatures (heat waves or cold spells) as a potential threat, despite evidence that extreme temperatures resulting from climate change have a significant adverse impact in Hong Kong.^12^^,^^13^

When asked to compare Hong Kong’s population’s awareness and preparedness level with other cities globally, 57.1% (n=545) regarded the Hong Kong community as having lower disaster awareness. Almost half (49.6%, n=462) of interviewees believed that disaster preparedness in Hong Kong was worse than other major cities.

**Socio-demographic predictors of perceptions on susceptibility to disasters, disaster awareness and preparedness**

Table 2 provides an overview of risk perception and self-reported health emergency and disaster preparedness at both individual and household levels in Hong Kong. Among all respondents, only 26.1% (n=261) reported having ever received some form of first aid training. For household-based preparedness, basic supplies such as face-masks, band-aids and basic medication such as anti-pyretic drugs were reported available in over 90% of interviewed households. In contrast, ownership of a first aid kit (49.4%, n=494) and non-perishable food and drinking water (57.4%, n=574) were less commonly reported. Among those reported to be chronic disease patients, 89% (n=189) had prepared long-term medications at home that could support their chronic illness for at least two weeks. In addition, although fire hazard was perceived as the most threatening disaster in Hong Kong, only 11.5% (n=114) of the respondents possessed fire extinguisher in the household. Overall, 75.1% (n=753) of the respondents were practicing at least three out of the five preparedness measures (equipped with a first aid kit, basic aids supplies, emergency food and drinking water, basic medication, and a fire extinguisher).

**Figure:**
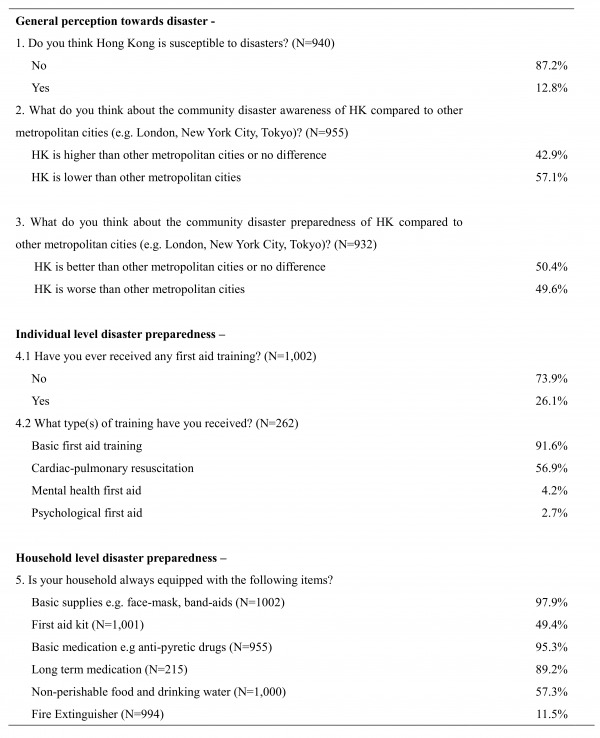
**Table 2. Overview of risk perception and self-reported disaster preparedness at individual and household level in Hong Kong**

Table 3 presents the results from multivariate Logistic Regression analyses on the predictive factors for disaster-related perceptions as well as actual disaster preparedness at both individual and household levels. Regarding the perceived susceptibility to disaster of Hong Kong, older age (65 years and above) was associated with a higher likelihood of perceiving Hong Kong as susceptible to disaster (OR=2.55; 95%CI=1.49-4.36, p<0.05) when compared to the younger age group (15-39 years). On the other hand, regarding the perceived disaster awareness of Hong Kong compared to other metropolitan cities, having attained post-secondary education and above (OR=1.92; 95%CI=1.21-3.05, p<0.001), living in the New Territories (OR=1.61; 95%CI=1.12-2.32, p<0.05), and being religious (OR=1.39; 95%CI=1.02-1.90, p<0.05) were associated with a higher likelihood of perceiving Hong Kong to have lower disaster awareness, while living with persons <15 yrs and >60 yrs (OR=72; 95%CI=0.54-0.96, p<0.05) and being married (OR=0.69; 95%CI=0.50-0.95, p<0.05) were associated with a lower likelihood of perceiving Hong Kong to have lower disaster awareness.

**Figure:**
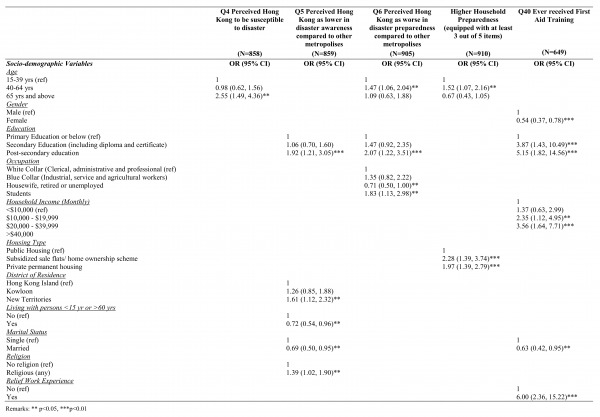
**Table 3. Multivariate Logistic Regression analyses on the socio-demographic predictors for: i) perceived susceptibility of Hong Kong to disasters, ii) perceived disaster awareness of Hong Kong compared to other metropolitan cities, iii) perceived disaster preparedness of Hong Kong compared to other metropolitan cities, and iv) actual household preparedness.**

Middle age (40-64 years) (OR=1.47; 95%CI=1.06-2.04, p<0.05), having attained post-secondary education or above (OR=2.07; 95%CI=1.12-3.51, p<0.05) and being students (OR=1.83; 95%CI=1.13-2.98, p<0.05) were associated with a higher likelihood of perceiving Hong Kong being worse in terms of disaster preparedness compared to other metropolitan cities, while being housewives, retired or unemployed (OR=0.71; 95%CI=0.50-1.00, p<0.05) was associated with a lower likelihood of such perception. In addition, higher education such as secondary education (OR=3.87; 95%CI=1.43-10.49, p<0.01) and post-secondary education (OR=5.15; 95%CI=1.82-14.56, p<0.01), higher monthly household income ($20,000-$39,999: OR=2.35, 95%CI=1.12-4.95; $40,000 and above: OR=3.56, 95%CI=1.64-7.71, p<0.01), and experience in relief work (OR=6.00, 95%CI=2.36-15.22, p<0.01) were associated with an increased likelihood of receiving first aid training while female (OR=0.54; 95%CI=0.37-0.78, p<0.01) and being married (OR=0.63; 95%CI=0.42-0.95, p<0.05) were associated with a decreased likelihood of receiving first aid training. Further, regarding the actual household preparedness, living in subsidized sale flats/ home ownership scheme (OR=2.25; 95%CI=1.40-3.63, p<0.01) and private permanent housing (OR=2.01; 95%CI=1.44-2.81, p<0.01) were associated with a higher likelihood of better household preparedness compared to those living in public housing. Moreover, the middle age group (40-64 years) (OR=1.52; 95%CI=1.07, 2.16 p<0.05) was associated with a higher likelihood of better household preparedness compared to the younger age group (15-39 years).

## DISCUSSION

**Perceptions on disaster risk, disaster awareness and disaster preparedness in Hong Kong**

The study examined the perception towards disasters in a population-dense urbanized community. Hong Kong is frequently affected by different hazards. Severe infectious disease outbreaks happen once every several years (i.e. Severe acute respiratory syndrome (SARS) in 2003^14^, swine flu in 2009^15^, and the reoccurring avian flu from multiple genotypes since 1997^16^^,^^17^). Typhoons strike the city 4-7 times during the annual rainy season.^18^ Fire accidents threaten life and cause damage and loss, for example, claiming 24 lives and causing 309 injuries in 2014.^19^ Extreme weather events, which have been documented to cause excess mortality^20^^,^^21^, have increased in recent years due to global climate change.^22^

However, urban public perceptions towards disasters appear to be disassociated from the actual occurrence of extreme events in this Chinese city. Only 12.8% people regarded Hong Kong as susceptible to disasters, indicating a low-level of disaster-related risk perceptions within the Hong Kong community. The majority (57.1%) of the respondents perceived themselves to have lower disaster awareness than other major metropolitan cities. The findings reflect the intuitive judgment of risk by lay people, which are based on a hazard’s catastrophic potential (the dread factor), uncertainty about the hazard (the unknown factor), and the number of people exposed to the risk.^23^

In addition, the majority of the community (nearly 80% of the respondents) reported having no experience of disasters, which could be demonstrating a gap in the application of the disaster concept to real life events. Since the adverse effects of disasters on individuals and households vary based on the nature of the disaster and personal circumstances, most people may have experienced of the strength (i.e. typhoon) and panic (i.e. infectious diseases) caused by a disaster but not experienced any personal harm or loss of personal property. This may cause them to not associate the word “disaster” with their perception of the extreme events that happened in their community.

When asked to choose the most threatening disaster to Hong Kong, 74% of respondents perceived infectious disease outbreaks to be the most threatening. Typhoons and fires were considered to be the most threatening by 12.9% and 7.3% of the respondents, respectively. These results are in line with Hong Kong’s exposure level to the different disasters. The 2003 SARS outbreak in Hong Kong had a high fatality rate of 17% 24 and caused a large disruption in people’s daily routines, resulting in a heightened risk awareness and concern for infectious disease outbreaks among the Hong Kong community. Timely weather warnings from Hong Kong Observatory and policies affecting work and school surround the occurrence of typhoons and the news media broadcast the damage of typhoons and fires to the public.

The survey found that Hong Kong people, 99.2% of the community, obtain information related to their perceived most threatening disaster from at least one source and 68.2% would refer to more than one source. Moreover, 73.2% of the respondents have heard of related measures to manage those disasters in Hong Kong. However, despite having the knowledge and information about threatening disasters, there is a gap between the sufficient information and the awareness of Hong Kong’s susceptibility to disasters. In other words, an individual does not personalize the risk of a disaster, even though the disaster is the most threatening from his/her perspective. Such a gap may also explain the pattern of preparedness in the following discussion.

**Household emergency and disaster health risk preparedness**

About half (49.6%) of the respondents perceived Hong Kong having a lower level of disaster preparedness compared to other metropolitan cities. We surveyed the following 6 household stock-piling items that are of importance in disaster preparedness: first aid kit, basic aids supplies, storage of food and water, basic medications, long-term medication and fire-extinguishers. (Table 2) Less than half of the respondents reported to keep a first aid kit at home, which was lower than previous findings from a 2010 Hong Kong study reporting first aid kits in 60.6% of families with young children. 25Most respondents reported to have the basic aids supplies (facemask and band-aid) and basic medication (anti-pyretics). Only 57.7% of the respondents stored extra food and water at home for emergencies. Long-term medication was found to be well-prepared by those in need, as almost 90% of people with chronic diseases (n=215) reported to have enough for at least two weeks. Although only a few people reported to have fire extinguishers at home, over 90% of interviewees lived in public, subsidized or private estates where fire extinguisher facilities should already be installed in compliance with the Hong Kong Fire Safety Ordinance^26^. This may not be applied to people living in temporary shelter, sublet rooms or partitioned flats, which often lack compliance with the Hong Kong Fire Safety Ordinance.

The analysis of adequate household preparedness for emergency and disasters among the Hong Kong community indicated that Hong Kong’s residents are inadequately prepared for disaster. Only 28.3 % of the respondents had all five items for household disaster preparedness. (Long-term medication was excluded, as the item is only applicable to people with chronic diseases and not to the whole community. In addition, both having a fire extinguisher at home and living in a residence equipped with government-approved fire extinguisher facilities were considered to fulfill the preparedness of fire extinguishers.) An Australian study by Cretikos et al. found similar results when investigating household disaster preparedness and information sources used before and during a disaster in New South Wales^27^. The affected community was poorly prepared, as less than one-fifth of the households had all items required for preparedness: a torch, battery operated radio, appropriate batteries, mobile phone, emergency contact list and first aid kit.^27^ Another study conducted by Bethel et. al. yielded similar results in the United States.^28^ Only 42.4% of households had all four preparedness items, which included three-day supply of drinking water and nonperishable food, a battery-operated radio with working batteries, and a flashlight with working batteries^28^. Their multivariate analysis indicated that respondents with poorer reported health status, disabilities, and multiple chronic diseases were less likely to prepare emergency supplies, yet they were more likely to be equipped with a three-day supply of medication^28^, which partly echoes our results that people with chronic diseases were more likely to be prepared with adequate long-term medication.

An inverse association was found in the elderly (65 years and above) between disaster risk perception and household preparedness compared with the younger age group (15-39 years old). The elderly had a significantly higher disaster risk perception (OR=2.55), which may result from having more experiences of casualty-inducing-disasters in their younger years when Hong Kong’s development, infrastructure and government measures were less well-established^29^. However, despite the higher risk perception, they have lower (OR=0.67) household preparedness than the younger age group. These results were contrary to previous studies conducted in North America and Japan, as older age was a significant predictors of higher preparedness levels^30^^,^^31^^,^^32^^,^^33^^,^^34^. This implies the need for further research and increasing health promotion for disaster preparedness among the elderly population.

In contrast to those with lower education levels, higher education (post-secondary education or above) was associated with a higher likelihood of perceiving Hong Kong to be worse than other metropolitan cities in terms of disaster awareness and preparedness. A similar trend was observed among those with secondary education or above, although such observations were not found to be statistically significant. One possible explanation for such trend may be that people with higher education are more conscious about disaster-related news and information globally. Muttarak conducted a survey among areas receiving tsunami warnings after the 2012 Indian Ocean earthquake in Thailand, and found that formal education can increase disaster preparedness and reduce vulnerability to natural hazards^35^. Also, higher education were correlated with better emergency supplies in studies done by Schultz^36^ and Eisenmen et al^37^, though no association was found between education level and the actual household preparedness in this study, possibly due to study limitations.

**Barriers for household disaster preparedness**

Although causes of household disaster unpreparedness were not examined in detail in this study, the literature suggests that lack of time and resources, lack of knowledge on how to achieve preparedness, and poor familiarity with preparedness items were common barriers to personal preparedness^38^^,^^39^. One study that investigated Hong Kong families with young children reported that a nearby convenience store or supermarket (13.6%), lack of storage space at home (12.1%) and not seeing the need to store items at home (6.6%) were commonly cited reasons for unpreparedness among the head of households^25^. Further studies in Chinese communities will be worthwhile to explore the gap between perceived and real vulnerabilities, actual preparedness practices, and potential areas for strengthening health education.

**Implications on promotion of disaster health risk literacy**

Disaster-related risk perception, according to our study, was not a predicting factor for individual or household level disaster preparedness. Although community disaster experiences appear to be positively correlated with preparedness, at both individual and household levels, the associations appear to be inconsistent. Both of the above arguments highlight that it is necessary not only to enhance emergency preparedness measures taken by each household, but also to promote awareness of the population’s vulnerability towards potential disasters in Hong Kong.

The study findings implies the insufficiency of the urban Chinese communities regarding disaster preparedness, which also calls for a reflection of the need for increasing disaster risk literacy^9^^,^^40^ among the general public. The investigation of the current study on the predicting factors for disaster risk perceptions and disaster preparedness provide strong insights for disaster health risk communications. Further studies on the predisposing, enabling and reinforcing factors related to disaster health risk literacy^9^ and preparedness will be important to inform policy makers on the key determinants for building community resilience towards health-related emergencies in the event of a disaster.

**Limitations**

A number of limitations should be noted. The first is the methodological limitations inherent in the use of a telephone survey. Households that do not possess a landline telephone service were likely missed. Nonetheless, among the Hong Kong community, almost all households have a landline telephone service. Other local published studies^11^ have also used this method and found reliable estimations on community behavior related responses in emergency crises. Second, a self-reported questionnaire was used to determine household preparedness, which may differ from observed measures of behaviors. Third, our findings may represent under-reported rates of preparedness because respondents might not have recognized the term “disaster kit” or “emergency kit” as used in the interview.

## CONCLUSION

The current findings suggest that future research and policy formulation should focus on strengthening household disaster preparedness, and enhancing disaster health risk literacy in the general public. High level disaster-related mitigation knowledge within the community is an essential asset in the development of preparedness enhancement programs. Further investigation will be necessary to examine other aspects of disaster health risk literacy, including the capability and skills to acquire, evaluate and comprehend disaster-related information, and the ability to implement emergency response plans to mitigate the impacts of disasters.

## COMPETING INTERESTS

The authors declare that no competing interests exist.
